# Supplementary material for: Immunological Signatures in Blood and Urine in 80 Individuals Hospitalized during the Initial Phase of COVID-19 Pandemic with Quantified Nicotine Exposure
Source: Int J Mol Sci. 2024 Mar 27;25(7):3714. doi: 10.3390/ijms25073714 (PMC11011256; doi:10.3390/ijms25073714)
Supplement: Supplementary file 1 [file ijms-25-03714-s001.zip › ijms-2828980-supplementary.pdf]

Supplemental Material #S1

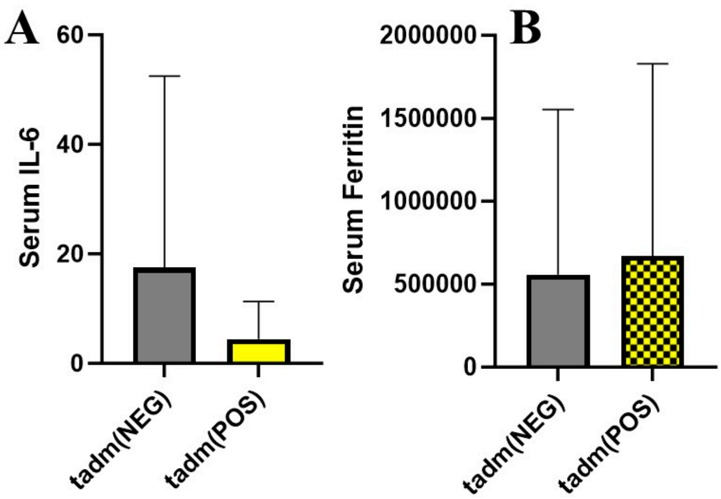

There was no difference in markers of non-specific inflammation, including IL-6 (**A**) and ferritin (**B**), between cotinine-positive and negative individuals.

Supplemental Material #S2

d-Cohen statistics for the urine and blood analyte with statistically significant difference between patient testing positive for cotinine vs cotinine negative individuals.

Blood

			95% Confidence Interval	
	Time Point	d Cohen's	Lower	Upper
MIC-A/B	tadm	0.87	-0.01	1.74
CD28	t48hrs	-0.90	-1.84	0.05
IFNγ	t48hrs	0.84	-0.10	1.77
MIC-A/B	t48hrs	1.10	0.14	2.05
MCP-3	t48hrs	1.14	0.17	2.10
CD8A	t48hrs	1.15	0.18	2.10
IL12	t48hrs	1.16	0.19	2.12
GZMB	t48hrs	1.25	0.27	2.21
CCL19	t48hrs	1.32	0.34	2.29
CD28	t7d	0.92	-0.20	2.02

Urine

			95% Confidence Interval	
	Time Point	d Cohen's	Lower	Upper
CCL20	tadm	-1.36	-2.23	-0.47
CD8A	tadm	-1.11	-1.96	-0.24
CXCL5	tadm	-1.07	-1.92	-0.20
MUC-16	tadm	-1.03	-1.88	-0.17
IL-12	tadm	-0.86	-1.70	-0.01
GZMH	tadm	-0.85	-1.68	0.00
TNFSF14	tadm	-0.81	-1.64	0.04
CCL20	t48hrs	0.16	-0.74	1.05
TRAIL	t48hrs	0.74	-0.18	1.65
ADA	t7d	-1.25	-2.41	-0.06
EGF	t7d	-1.10	-2.24	0.07

Supplemental Material #S3

List of all analytes tested:

Adenosine deaminase (ADA)
Adhesion G-protein coupled receptor G1 (ADGRG1)
Angiopoietin-1 (ANGPT1)
Angiopoietin-1 receptor (TEK)
Angiopoietin-2 (ANGPT2)
Arginase-1 (ARG1)
C-C motif chemokine 13 (CCL13)
C-C motif chemokine 17 (CCL17)
C-C motif chemokine 19 (CCL19)
C-C motif chemokine 2 (CCL2)
C-C motif chemokine 20 (CCL20)
C-C motif chemokine 23 (CCL23)
C-C motif chemokine 3 (CCL3)
C-C motif chemokine 4 (CCL4)
C-C motif chemokine 7 (CCL7)
C-C motif chemokine 8 (CCL8)
C-X-C motif chemokine 10 (CXCL10)
C-X-C motif chemokine 11 (CXCL11)
C-X-C motif chemokine 13 (CXCL13)
C-X-C motif chemokine 5 (CXCL5)
C-X-C motif chemokine 9 (CXCL9)
CD27 antigen (CD27)
CD40 ligand (CD40LG)
CD70 antigen (CD70)
CD83 antigen (CD83)
Carbonic anhydrase 9 (CA9)
Caspase-8 (CASP8)
Cytotoxic and regulatory T-cell molecule (CRTAM)
Decorin (DCN)
Fibroblast growth factor 2 (FGF2)
Fractalkine (CX3CL1)
Galectin-1 (LGALS1)
Galectin-9 (LGALS9)
Granzyme A (GZMA)
Granzyme B (GZMB)
Granzyme H (GZMH)
Growth-regulated alpha protein (CXCL1)
Heme oxygenase 1 (HMOX1)

Hepatocyte growth factor (HGF)
ICOS ligand (ICOSLG)
Interferon gamma (IFNG)
Interleukin-1 alpha (IL1A)
Interleukin-10 (IL10)
Interleukin-12 (IL12A_IL12B)
Interleukin-12 receptor subunit beta-1 (IL12RB1)
Interleukin-13 (IL13)
Interleukin-15 (IL15)
Interleukin-18 (IL18)
Interleukin-2 (IL2)
Interleukin-33 (IL33)
Interleukin-4 (IL4)
Interleukin-5 (IL5)
Interleukin-6 (IL6)
Interleukin-7 (IL7)
Interleukin-8 (CXCL8)
Killer cell immunoglobulin-like receptor 3DL1 (KIR3DL1)
Lymphocyte activation gene 3 protein (LAG3)
Lysosome-associated membrane glycoprotein 3 (LAMP3)
MHC class I polypeptide-related sequence A and MHC class I polypeptide-related sequence B (MIC A/B)
Macrophage colony-stimulating factor 1 (CSF1)
Macrophage metalloelastase (MMP12)
Matrilysin (MMP7)
Mucin-16 (MUC16)
Natural cytotoxicity triggering receptor 1 (NCR1)
Natural killer cell receptor 2B4 (CD244)
Natural killer cells antigen CD94 (KLRD1)
Nitric oxide synthase, endothelial (NOS3)
Placenta growth factor (PGF)
Platelet-derived growth factor subunit B (PDGFB)
Pleiotrophin (PTN)
Pro-epidermal growth factor (EGF)
Programmed cell death 1 ligand 1 (CD274)
Programmed cell death 1 ligand 2 (PDCD1LG2)
Programmed cell death protein 1 (PDCD1)
Stromal cell-derived factor 1 (CXCL12)
T-cell surface glycoprotein CD4 (CD4)
T-cell surface glycoprotein CD5 (CD5)
T-cell surface glycoprotein CD8 alpha chain (CD8A)
T-cell-specific surface glycoprotein CD28 (CD28)

Transforming growth factor beta-1 proprotein (TGFB1)
Tumor necrosis factor (TNFa)
Tumor necrosis factor ligand superfamily member 10 (TNFSF10)
Tumor necrosis factor ligand superfamily member 12 (TNFSF12)
Tumor necrosis factor ligand superfamily member 14 (TNFSF14)
Tumor necrosis factor ligand superfamily member 6 (FASLG)
Tumor necrosis factor receptor superfamily member 12A (TNFRSF12A)
Tumor necrosis factor receptor superfamily member 21 (TNFRSF21)
Tumor necrosis factor receptor superfamily member 4 (TNFRSF4)
Tumor necrosis factor receptor superfamily member 5 (CD40)
Tumor necrosis factor receptor superfamily member 9 (TNFRSF9)
Vascular endothelial growth factor A (VEGFA)
Vascular endothelial growth factor receptor 2 (KDR)
